# Different Phenotypes in Monozygotic Twins, Carriers of the Same Pathogenic Variant for Hypertrophic Cardiomyopathy

**DOI:** 10.3390/life12091346

**Published:** 2022-08-30

**Authors:** Manuel Rodríguez Junquera, María Salgado, Francisco González-Urbistondo, Alberto Alén, José Julián Rodríguez-Reguero, Iria Silva, Eliecer Coto, Pablo Avanzas, César Morís, Juan Gómez, Rebeca Lorca

**Affiliations:** 1 Servicio de Cardiología, Clínica Universitaria de Navarra, 31008 Pamplona, Spain; 2 Heart Area, Hospital Universitario Central de Asturias, 33011 Oviedo, Spain; 3 Unidad de Referencia de Cardiopatías Familiares-HUCA, Área del Corazón y Departamento de Genética Molecular, Hospital Universitario Central de Asturias, 33011 Oviedo, Spain; 4 Instituto de Investigación Sanitaria del Principado de Asturias (ISPA), 33011 Oviedo, Spain; 5 Redes de Investigación Cooperativa Orientadas a Resultados en Salud (RICORs), 28029 Madrid, Spain; 6 CIBER-Enfermedades Respiratorias, 28029 Madrid, Spain; 7 Universidad de Oviedo, 33006 Oviedo, Spain

**Keywords:** hypertrophic cardiomyopathy (HCM), inherited cardiomyopathies, identical twins

## Abstract

Hypertrophic cardiomyopathy (HCM) is a monogenic disease with autosomal dominant inheritance. Genotype–phenotype relationships are complex, with variable penetrance even within the same family. The involvement of other modulating genetic and environmental factors is unknown. We aimed to analyze the HCM in monozygotic twins, carriers of the same founder pathogenic variant *MYBPC3* p.G263*. The relationship was verified using the PowerPlex 16 HS System kit. Phenotypic differences and environmental differences (overloading conditions, coexistence and location, lifestyle, sport, and intensity) were analyzed. Three pairs of twins genetically identical for all markers and carriers of *MYBPC3* G263* were identified. No environmental differences were identified. One of the 89-year-old twins had symptomatic severe obstructive HCM that required septal ablation, while her twin has remained asymptomatic with mild phenotype >80 years. A 49-year-old twin had a severe phenotype of obstructive HCM and pending myectomy, while his twin had a mild asymptomatic phenotype. In the last pair of twins, one presented a much larger left ventricular hypertrophy than his identical twin. In summary, we present three pairs of HCM twin patients sharing not only the genetic cause of the inherited disease but the entire genetic background. Despite identical genetic information and the absence of other known clinical, environmental, or lifestyle differences, the severity of the HCM phenotype is strikingly different. These unexplained differences should prompt the study of other unknown modulating factors, either epigenetic or environmental.

## 1. Introduction

Hypertrophic Cardiomyopathy (HCM) is the most common inherited heart muscle disease, with a reported prevalence that ranges from 1:200 to 1:500 [1]. HCM has been defined by the presence of increased left ventricular (LV) wall thickness that is not solely explained by abnormal loading conditions. About two-thirds of patients with HCM present (or develop over time) obstruction to the LV outflow tract (LVOTO), either only during maneuvers that change loading conditions and LV contractility or at rest [2,3,4,5]. However, a third of HCM patients remain nonobstructive [6]. Besides the LVOTO, systolic dysfunction and potential malignant arrhythmias related to sudden cardiac death (SCD) are considered the most feared complications related to HCM [1]. 

HCM is inherited in an autosomal dominant pattern. In fact, up to 60% of patients with HCM have an identifiable pathogenic or likely pathogenic variant, considered disease causing [1,7]. In this sense, most pathogenic variants are identified in sarcomeric genes: beta myosin heavy chain 7 (*MYH7*) and myosin-binding protein C3 (*MYBPC3*) [6,8,9,10,11,12]. Nonetheless, to date, the precise mechanisms by which HCM pathogenic variants result in the clinical phenotype have not been fully elucidated. In fact, it has been suggested that sarcomeric proteins may not be solely responsible [6].

In those relatives who are carriers of the same pathogenic variant, the likelihood of developing clinical HCM is considered high but also age dependent [6,13]. However, the age at which HCM expression may occur and its clinical expression are variable [6]. This highlights the hypothesis that other unknown non-inherited factors may influence the clinical course of the disease.

Genotype–phenotype correlations have been inconsistent. In general, because of the large number and diversity of HCM-pathogenic variants, the genotype cannot be used to anticipate the individual clinical expression [6]. In fact, there is a wide range of clinical expression in HCM: from almost asymptomatic to SCD [1]. What is more, the clinical and phenotypical variability is not only present among different patients harboring different pathogenic variants but also within the same family sharing the genetic background responsible for the HCM. 

In this sense, some studies of founder pathogenic variants in *MYBPC3* have provided an opportunity to better define their clinical profiles and genotype–phenotype correlations and analyze possible non-inherited factors [13,14,15,16,17,18,19,20,21]. In this regard, the founder pathogenetic variant *MYBPC3* p.G263* was reported to share with most truncating pathogenic variants in this gene a late onset, a relatively benign clinical course in the young, and high penetrance [13]. Moreover, monozygotic twins also provide a privileged scenario to analyze the clinical differences in HCM manifestations, despite the identical backgrounds. 

The aim of this study was to describe the clinical differences in three couples of twins with HCM, carriers of the same founder pathogenic variant *MYBPC3* p.G263*. 

## 2. Materials and Methods

### 2.1. Study Population

In this retrospective clinical study, we reviewed all patients labeled as “twins” in their medical records and referred them for genetic testing with familiar HCM diagnoses. We identified all suspected twins who were carriers of the *MYBPC3* p.Gly263Ter (c.787G > T: NM_000256.3) as reported elsewhere [13]. 

Clinical and genetic screening in all available relatives was systematically performed. All patients who wished to participate signed written consent to grant access to their genetic data for investigational purposes, and the research protocol followed institutional ethics guidelines. This study was evaluated by the local Ethical Committee (CEImPA 2022.350).

Clinical data and demographic information were investigated, recording the personal and family history of symptoms, physical activity, lifestyle, clinical situations that may produce overloading conditions, arrhythmic events, electrocardiographic parameters, possible device implantations and therapies, LVOT management, and medical treatment. In the youngest twins, echocardiograms were updated for simultaneous phenotypical evaluation and analyzed by an imaging expert cardiologist, blind to their clinical and genetic status. For the older twins, the last echocardiograms were reviewed. Cardiac magnetic resonance (CMR) parameters, if available, were also analyzed.

### 2.2. Genetic Analysis 

Blood samples were obtained from all patients who agreed to undergo genetic testing, collected in a 9 mL tube with EDTA anticoagulant. We isolated DNA from their peripheral blood leukocytes by standard salting-out method, a simple and nontoxic DNA extraction technique that isolates high-quality DNA from the whole blood [22]. Genetic testing was carried out from DNA samples from all referred patients. 

Index patients referred for genetic testing with familiar HCM diagnosis were studied for the main sarcomere genes by next-generation sequencing, as reported by Gómez et al. [23,24]. In the oldest cases, only nine genes were evaluated (Supplementary Table S1). However, in the most recent ones, 195 genes were included (Supplementary Table S2). These genes were sequenced with the Ion Torrent technology that uses semiconductor chips and the Ion GeneStudio S5 Sequencer (Termo Fisher Scientific). The raw data were processed with the Torrent Suite v5 software. Reads assembling and variant identification were performed with the Variant Caller (VC). Ion Reporter (Thermo Fisher Scientific, Waltham, MA, USA) and HD Genome One (DREAMgenics S.L., Oviedo, Spain) software were used for variant annotation, including population, functional, disease-related and in silico predictive algorithms. The Integrative Genome Viewer (IGV, Broad Institute, Cambridge, MA, USA) was used for the analysis of depth coverage, sequence quality, and variant identification. Interpretation of all gene variants with an allele frequency < 0.01 was based on the American College of Medical Genetics and Genomics (ACMG-AMP) 2015 Standards and Guidelines [25]. 

Familial screening for *MYBPC3* p.G263* variant was performed by sanger sequencing in an ABI3130XL sequencer (Thermo Fisher Scientific, Waltham, MA, USA). (Supplementary Figure S1).

In order to verify that all patients included were monozygotic twins, the PowerPlex 16 HS System kit was used. This kit consists of 16 microsatellite markers so that it coamplifies loci D18S51, D21S11, TH01, D3S1358, Penta E (labeled with fluorescein); FGA, TPOX, D8S1179, vWA, and amelogenin (TMR-labelled); CSF1PO, D16S539, D7S820, D13S317, D5S818, and Penta D (labeled JOE). Only identical twins—identical for all markers—were included in this study. 

## 3. Results

Three pairs of twins were carriers of the same founder pathogenic variant *MYBPC3* p.G263*. *MYBPC3* p.G263* is a founder pathogenic variant in our region as reported elsewhere [13], according to ACMG-AMP criteria [25]. It is a truncating variant in a gene in which loss of function is a known mechanism of disease. Computational evidence supports a deleterious effect on the gene. It is absent from controls in the Exome Sequencing Project, 1000 Genomes Project, and ExAC databases. The robust segregation information in multiple affected family members strongly supports its pathogenicity [13].

**Figure 1:**
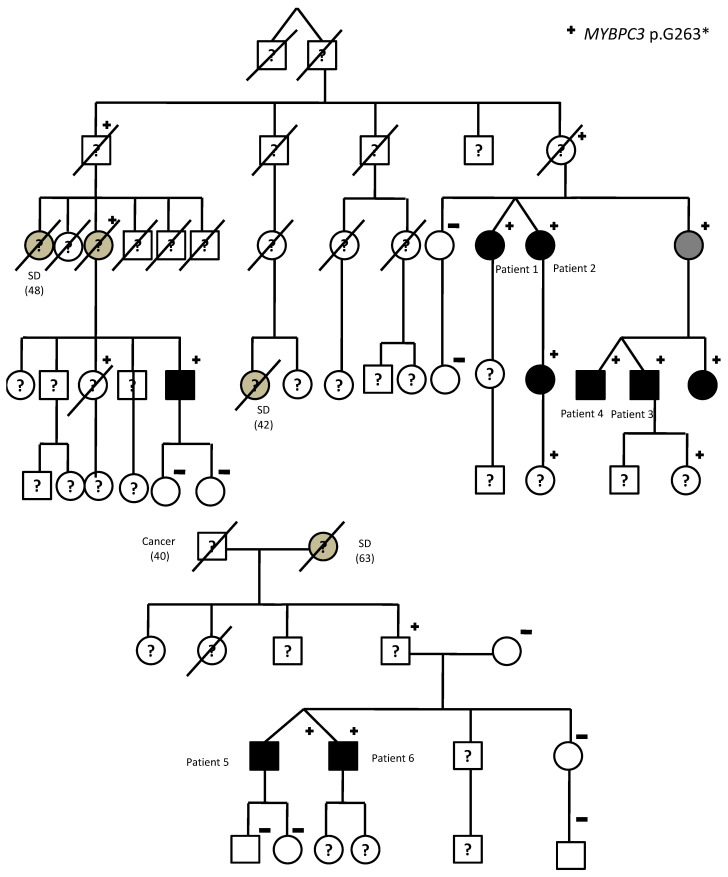
*MYBPC3* p.G263* twin carriers: Family pedigrees; SD—sudden death; age of deceased patients due to SD is shown in brackets. Symbols denote sex and disease status: “+”—carriers; “−”—noncarriers; without sign—genetic status unknown; box—male; circle—female; black darkened—hypertrophic cardiomyopathy phenotype (HCM); grey darkened—unexplained SD or suspected but not confirmed HCM; symbol clear—negative phenotype; “?”—unknown phenotype; slashed—deceased.

We identified two female twins aged 89 years (patients 1 and 2), two 49-year-old men (patients 3 and 4), and two 47-year-old men (patients 5 and 6) monozygotic twins. Family pedigrees are shown in Figure 1. The first family (including patients 1 to 4) was first reported by Lorca et al. [13] and expanded in this pedigree. The second family (patients 5 and 6) was diagnosed afterward. 

The three pairs of twins were monozygotic twins, genetically identical for all analyzed markers. The most important clinical characteristics are summarized in Table 1. 

There were no geographical differences in their lifetime. They all lived together for at least 18 years, and later, they spent most of their lives in nearby locations. No patient was hypertensive. Physical activity levels and lifestyle were similar between twins. Both women had only one pregnancy. There were no other evident differences that may justify alternative abnormal loading conditions among twins. 

**Figure 2:**
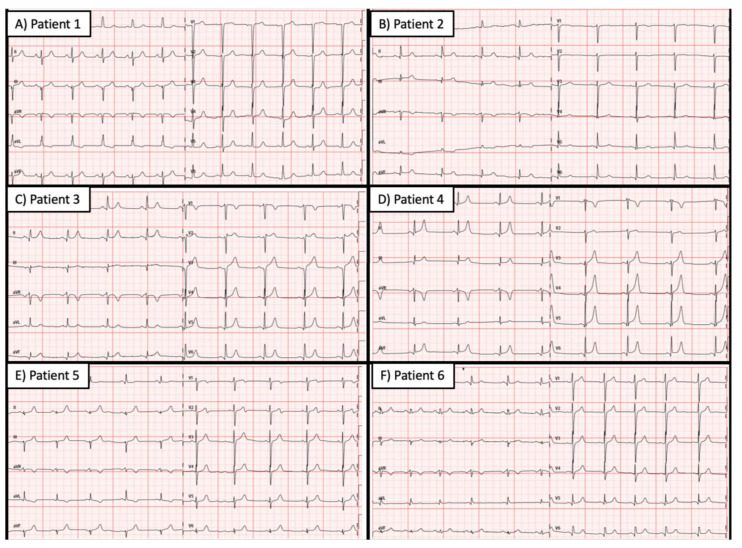
Electrocardiograms from identical twins, carriers of the founder pathogenic variant *MYBPC3* p.G263*. (**A**) Patient 1–89 years old woman with obstructive hypertrophic cardiomyopathy (HCM) after septal ablation. (**B**) Patient 2–89 years old woman with mild HCM. (**C**) Patient 3–49 years old men with severe obstructive HCM. (**D**) Patient 4–49 years old men with mild HCM. (**E**) Patient 5–47 years old men with severe HCM. (**F**) Patient 2–47 years old men with mild HCM.

Electrocardiograms (ECGs) were similar between male twins (3–4 and 5–6). ECGs from twins 1 and 2 showed differences that can be explained by septal ablation (Figure 2).

However, HCM phenotype expression was different between twins. Patient 1 presented a very symptomatic phenotype of HCM due to severe LVOT. She required septal ablation that was clinically effective and nowadays remains paucisymptomatic at the age of 89 years old. In contrast, her identical twin (patient 2) presented a borderline phenotype with a maximal LV thickness of 13 mm, above 80 years old. 

Likewise, patient 3 is a 49-year-old twin with a severe and very symptomatic phenotype of obstructive HCM. Moreover, patient 3 presented with atrial fibrillation that significatively worsened his symptoms. An invasive procedure with myectomy is currently being evaluated. Conversely, her identical twin brother remains completely asymptomatic, with a mild phenotype of HCM. 

Finally, neither of the younger twins present LVOT. Nonetheless, maximal LV thickness is significantly higher in patient 5 than in his identical twin brother, measured by a simultaneously performed echocardiogram. CMRs were also performed, but one year apart. LV thickness measured by CRM was higher than by echocardiogram. This could be explained by a suboptimal image quality due to a limited acoustic window. However, the differences persisted. CMR showed a severe LV hypertrophy of 28 mm in patient 5 versus 19 mm in patient 6. 

## 4. Discussion

In general, HCM is inherited as an autosomal dominant genetic trait. Subsequently, there is a 50% risk of transmission to offspring [1]. The genetic basis of HCM has been widely studied during the past decades. In fact, genetic sequencing of the main sarcomere genes enables the identification of a definite causative genetic (pathogenic or likely pathogenic) variant in up to 60% of HCM patients [1].

Therefore, nowadays, genetic testing has become a key tool for family screening. In fact, when a definite disease-causing variant is identified in the proband, clinical evaluation is only recommended after the positive genetic screening. Only those relatives who were carriers of the familial pathogenic variant may have the risk of developing the disease. 

However, because of the variability in clinical penetrance and phenotype severity of HCM, nowadays, the impact of genetic testing is limited beyond family screening. Thus, studying possible modifiers of the clinical phenotype in patients sharing the same genetic background may be useful to help understand these differences. 

In this scenario, the analysis of monozygotic twins (carrying not only the same disease-causing genetic variant but also sharing all genetic information) provides a privileged scenario to approach these concerns. 

Limited case series studies have tried to compare the clinical expression of the HCM disease in monozygotic twins with identical genetic information. On the one hand, some authors have reported great similarities between twins, highlighting the powerful genetic determinant for morphologic and clinical expression. In addition, they suggest a minor role of other nonhereditary modifier factors, such as environmental ones. In 2006, Zenovich et al. reported two identical twins diagnosed with HCM who developed apical aneurysm at the same age [26]. In 2015, Goh et al. reported two HCM monozygotic twins who both had a cardiac arrest at the same age [27]. Moreover, in 2020, Maron et al. described a remarkable concordance not only in phenotype findings but also in the clinical course of two male identical twins with obstructive HCM [28]. 

Contrary to these authors, in this study, we describe three pairs of monozygotic twins with a radically opposite progression of the disease between siblings. In each pair of twins, one of them has a more severe phenotype than his/her twin. In this sense, other studies have emphasized the importance of other nonhereditary factors. In 2021, Repetti et al. studied 11 pairs of monozygotic twins, finding clinical discordances in all of them, emphasizing the important role of environmental influence and epigenetics [29]. Similarly, Palka et al. [30] and Kovács et al. [31] reported two other isolated cases of identical twins with different clinical courses.

To our knowledge, this is the largest series reported to date, describing monozygotic twins harboring the same pathogenic variant associated with HCM. Interestingly, patients 1 and 2 are aunts of patients 3 and 4 (Figure 1). Moreover, as *MYBPC3* p.G263* is a founder pathogenic variant in our region, all carriers are considered to be long-related [13]. As previously reported, *MYBPC3* p.G263* shared with most truncating pathogenic variants in this gene a late onset, a relatively benign clinical course in the young, and high penetrance [13]. General clinical data of twins, carriers of *MYBPC3* p.G263* variant, are consistent with previous data (high penetrance, as they are all clinically affected, and relatively benign clinical course, without severe cardiac dysfunction, cardiac transplant, or sudden cardiac death). However, despite the identical genetic background between twins, their phenotype (the severity of hypertrophy and the presence of severe left ventricular outflow tract obstruction) is strikingly different. Moreover, the oldest couple of twins is nearly 90 years old. As a result, no other relevant age-related phenotypic differences are expected to develop.

As we were unable to identify possible differences in lifestyle or abnormal loading conditions that may influence genetic expression, additional studies are needed.

## 5. Study Limitations

Further clinical and epidemiologic studies in HCM monozygotic twins’ patients are required to identify those environmental factors that influence phenotypical manifestations of HCM. 

## 6. Conclusions

We presented three pairs of HCM monozygotic twins, patients sharing not only the genetic cause of the inherited disease but the entire genetic background. However, despite the identical genetic information and the absence of other known clinical, environmental, or lifestyle differences, the severity of the HCM phenotype is strikingly different. These unexplained differences should prompt the study of other unknown modulating factors, either epigenetic or environmental.

## Figures and Tables

**Table 1:** Clinical data and demographic information about the three pairs of monozygotic twins, carriers of the founder pathogenic variant MYBPC3 p.G263*. HCM—Hypertrophic cardiomyopathy phenotype; LV—left ventricle; LVOT—obstruction to the LV outflow tract.

Patient	1	2	3	4	5	6
Age (years old)	89	89	49	49	47	47
Phenotype	HCM with severe LVOT	Mild HCM	HCM with severe LVOT	Mild HCM	HCM	Mild HCM
Maximal LV thickness By echocardiogram (year of evaluation)	29 mm (2015)	13 mm (2014)	22 mm (2022)	15 mm (2022)	18 mm (2022)	13 mm (2022)
Arrhythmias	.	.	Atrial fibrillation	.	.	.
Worse symptoms	Dyspnea III/IV NYHA	.	Dyspnea III/IV NYHA	.	.	.
Life together	30 years	30 years	18 years	18 years	34 years	34 years
Physical activity	Field/farm work	Field/farm work	Intense Physical activity	Intense Physical activity	Intense Physical activity	Intense Physical activity
Hypertension	.	.	.	.	.	.
Pregnancies	1	1	.	.	.	.

## Data Availability

Not applicable.

## Supplementary Materials

The following supporting information can be downloaded at: https://www.mdpi.com/article/10.3390/life12091346/s1.
