# Supplementary material for: Different Phenotypes in Monozygotic Twins, Carriers of the Same Pathogenic Variant for Hypertrophic Cardiomyopathy
Source: Life (Basel). 2022 Aug 30;12(9):1346. doi: 10.3390/life12091346 (PMC9506491; doi:10.3390/life12091346)

Supplementary table S1: NGS panel sequenced in oldest index cases.

Gene	Chr	Exons	Amplicons	Total Bases	Covered Bases	Missed Bases	Overall Coverage
MYH7	chr14	38	52	6188	6116	72	0.988
MYBPC3	chr11	33	46	4146	4081	65	0.984
TNNT2	chr1	22	18	1081	1081	0	1.000
TNNI3	chr19	7	9	702	702	0	1.000
MYL2	chr12	7	7	571	571	0	1.000
MYL3	chr3	6	8	648	648	0	1.000
TPM1	chr15	16	19	1500	1490	10	0.993
ACTC1	chr15	6	10	1194	1109	85	0.929
TNNC1	chr3	6	8	546	546	0	1.000

Suplemmentary table S2: NGS panel sequenced in recent index cases (patient 5-6).

Name	Chr	Amplicons	Total Bases	Covered Bases	Missed Bases	Overall Coverage
ABCC8	chr11	47	5139	5139	0	1
ABCC9	chr12	44	5178	5178	0	1
ACTA2	chr10	8	1214	1214	0	1
ACTC1	chr15	8	1194	1194	0	1
ACTN2	chr1	28	3112	3112	0	1
ACVRL1	chr12	14	1602	1602	0	1
AKAP9	chr7	90	12224	12224	0	1
ANK2	chr4	93	12411	12411	0	1
ANKRD1	chr10	9	1050	1050	0	1
APOB	chr2	85	13982	13982	0	1
APOE	chr19	11	1232	1232	0	1
BAG3	chr10	12	1768	1768	0	1
BGN	chrX	12	1457	1457	0	1
BLK	chr8	21	2118	2118	0	1
BMP10	chr2	9	1375	1375	0	1
BMPR1A	chr10	13	1709	1709	0	1
BMPR1B	chr4	13	1709	1709	0	1
BMPR2	chr2	25	3247	3247	0	1
BRAF	chr7	23	2481	2481	0	1
CACNA1C	chr12	64	7626	7626	0	1
CACNA1D	chr3	64	7150	7150	0	1
CACNA2D1	chr7	51	5327	5327	0	1
CACNB2	chr10	26	3269	3269	0	1
CALM1	chr14	7	690	690	0	1
CALM2	chr2	7	664	664	0	1
CALM3	chr19	8	750	750	0	1
CALR3	chr19	14	1605	1574	31	0.9807
CASQ2	chr1	13	1750	1750	0	1
CAV3	chr3	4	476	476	0	1
CBL	chr11	23	2881	2881	0	1
CHRM2	chr7	8	1451	1451	0	1
CHST14	chr15	7	1181	1181	0	1
COL1A1	chr17	56	4905	4905	0	1
COL1A2	chr7	55	4621	4621	0	1
COL3A1	chr2	57	4911	4911	0	1
COL5A1	chr9	78	6256	6256	0	1
COL5A2	chr2	64	7200	7200	0	1
CRYAB	chr11	5	678	678	0	1
CSRP3	chr11	6	635	635	0	1
CTNNA3	chr10	29	3758	3758	0	1

CTNNB1	chr3	19	2486	2486	0	1
DES	chr2	15	1503	1503	0	1
DMD	chrX	106	12161	12161	0	1
DNAJC19	chr3	6	411	411	0	1
DOLK	chr9	9	1667	1667	0	1
DSC2	chr18	25	2912	2912	0	1
DSG2	chr18	28	3507	3507	0	1
DSP	chr6	57	8856	8856	0	1
DTNA	chr18	29	3795	3795	0	1
EIF2AK3	chr2	32	4201	4201	0	1
EIF2AK4	chr15	51	6900	6900	0	1
ELN	chr7	37	4094	4094	0	1
EMD	chrX	9	1005	1005	0	1
ENG	chr9	22	2753	2753	0	1
EYA4	chr6	26	3039	3039	0	1
FBN1	chr15	73	9266	9266	0	1
FHL1	chrX	11	1472	1466	6	0.9959
FHOD3	chr18	48	6319	6319	0	1
FKRP	chr19	11	1498	1498	0	1
FKTN	chr9	13	1509	1509	0	1
FLNA	chrX	76	10294	10294	0	1
FLNC	chr7	75	8658	8658	0	1
GAA	chr17	27	3049	3049	0	1
GATA4	chr8	12	1392	1392	0	1
GATA5	chr20	12	1494	1494	0	1
GATAD1	chr7	9	1060	1060	0	1
GCK	chr7	17	1608	1608	0	1
GDF2	chr10	7	1390	1390	0	1
GLA	chrX	10	1360	1360	0	1
GLIS3	chr9	22	3293	3293	0	1
GPD1L	chr3	11	1376	1376	0	1
HCN4	chr15	28	4012	4012	0	1
HFE	chr6	9	1125	1107	18	0.984
HNF1A	chr12	16	2017	2017	0	1
HNF1B	chr17	16	1856	1856	0	1
HNF4A	chr20	20	1860	1860	0	1
HRAS	chr11	5	683	683	0	1
ILK	chr11	14	1959	1959	0	1
JAG1	chr20	35	3917	3917	0	1
JPH2	chr20	17	2402	2402	0	1
JUP	chr17	21	2368	2368	0	1
KCNA5	chr12	11	1892	1892	0	1
KCND2	chr7	15	2193	2193	0	1
KCND3	chr1	17	2318	2318	0	1
KCNE1	chr21	2	400	400	0	1

KCNE2	chr21	2	382	382	0	1
KCNE3	chr11	2	362	362	0	1
KCNE5	chrX	3	479	479	0	1
KCNH2	chr7	28	4017	4017	0	1
KCNJ11	chr11	6	1183	1183	0	1
KCNJ2	chr17	8	1294	1294	0	1
KCNJ5	chr11	9	1340	1340	0	1
KCNJ8	chr12	8	1375	1375	0	1
KCNK3	chr2	9	1285	1285	0	1
KCNQ1	chr11	20	2206	2206	0	1
KLF11	chr2	11	1739	1739	0	1
KRAS	chr12	6	737	737	0	1
LAMA4	chr6	55	7540	7540	0	1
LAMP2	chrX	15	1626	1626	0	1
LDB3	chr10	24	2668	2664	4	0.9985
LDLR	chr19	21	2763	2763	0	1
LDLRAP1	chr1	14	1377	1377	0	1
LIPA	chr10	14	1650	1650	0	1
LMNA	chr1	18	2369	2369	0	1
LOX	chr5	13	1604	1604	0	1
LZTR1	chr22	29	2733	2733	0	1
MAP2K1	chr15	12	1292	1292	0	1
MAP2K2	chr19	13	1313	1313	0	1
MAT2A	chr2	11	1638	1638	0	1
MED12	chrX	59	6984	6984	0	1
MFAP5	chr12	9	972	972	0	1
MIB1	chr18	36	4071	4071	0	1
MURC	chr9	7	1195	1195	0	1
MYBPC3	chr11	35	4155	4155	0	1
MYH11	chr16	51	6391	6391	0	1
MYH6	chr14	51	6190	6190	0	1
MYH7	chr14	45	6188	6188	0	1
MYL2	chr12	7	571	571	0	1
MYL3	chr3	6	648	648	0	1
MYLK	chr3	41	6055	6055	0	1
MYLK2	chr20	21	2391	2391	0	1
MYOZ2	chr4	8	1045	1045	0	1
MYPN	chr10	36	4983	4983	0	1
NEBL	chr10	38	5002	5002	0	1
NEUROD1	chr2	6	1121	1121	0	1
NEXN	chr1	21	2628	2628	0	1
NF1	chr17	74	9161	9161	0	1
NKX2-5	chr5	9	1142	1142	0	1
NNT	chr5	37	4311	4306	5	0.9988
NOTCH1	chr9	63	8008	7987	21	0.9974

NOTCH3	chr19	51	7296	7296	0	1
NPPA	chr1	4	486	486	0	1
NRAS	chr1	5	610	610	0	1
PAX4	chr7	10	1482	1482	0	1
PAX6	chr11	13	2035	2035	0	1
PCSK9	chr1	16	2199	2199	0	1
PDLIM3	chr4	13	1733	1733	0	1
PDX1	chr13	7	952	952	0	1
PKP2	chr12	21	2786	2786	0	1
PLN	chr6	1	169	169	0	1
PLOD1	chr1	28	3325	3325	0	1
PPARG	chr3	12	1588	1588	0	1
PRDM16	chr1	37	4681	4654	27	0.9942
PRKAG2	chr7	19	1974	1974	0	1
PRKG1	chr10	26	3087	3087	0	1
PSEN1	chr14	12	1504	1504	0	1
PSEN2	chr1	15	1447	1447	0	1
PTPN11	chr12	16	1936	1936	0	1
RAF1	chr3	18	2107	2107	0	1
RBM20	chr10	27	3824	3817	7	0.9982
RIT1	chr1	8	771	771	0	1
RYR2	chr1	134	15954	15954	0	1
SCN10A	chr3	49	7221	7221	0	1
SCN1B	chr19	9	1066	1066	0	1
SCN2B	chr11	7	848	848	0	1
SCN3B	chr11	8	898	898	0	1
SCN4B	chr11	7	887	887	0	1
SCN5A	chr3	44	6423	6423	0	1
SDHA	chr5	19	2145	2145	0	1
SGCD	chr5	8	1025	1025	0	1
SHOC2	chr10	14	1829	1829	0	1
SKI	chr1	18	2537	2537	0	1
SLC22A5	chr5	14	1856	1856	0	1
SLMAP	chr3	33	3799	3799	0	1
SMAD1	chr4	12	1698	1698	0	1
SMAD2	chr18	13	1504	1504	0	1
SMAD3	chr15	14	1452	1452	0	1
SMAD4	chr18	18	1769	1769	0	1
SMAD9	chr13	13	1704	1704	0	1
SNTA1	chr20	17	1918	1918	0	1
SOS1	chr2	40	4232	4232	0	1
SOS2	chr14	46	4919	4919	0	1
SPRED1	chr15	13	1405	1405	0	1
TAZ	chrX	14	1043	1043	0	1
TBX20	chr7	15	1748	1748	0	1

TBX4	chr17	16	2038	2038	0	1
TBX5	chr12	12	1637	1637	0	1
TCAP	chr17	5	524	524	0	1
TGFB2	chr1	12	1409	1409	0	1
TGFB3	chr14	10	1589	1589	0	1
TGFBR1	chr9	13	1614	1614	0	1
TGFBR2	chr3	12	1859	1859	0	1
TMEM43	chr3	13	1323	1323	0	1
TMPO	chr12	25	3385	3385	0	1
TNNC1	chr3	7	546	546	0	1
TNNI3	chr19	7	702	702	0	1
TNNI3K	chr1	30	3758	3710	48	0.9872
TNNT2	chr1	17	1081	1081	0	1
TPM1	chr15	15	1500	1500	0	1
TRDN	chr6	48	4418	4418	0	1
TRPM4	chr19	32	3895	3895	0	1
TTN	chr2	800	132581	129775	2806	0.9788
TTR	chr18	5	484	484	0	1
TXNRD2	chr22	23	2543	2543	0	1
VCL	chr10	36	4505	4505	0	1

Supplementary figure S1: Electropherograp of Sanger sequencing of *MYBPC3* p.Gly263Ter variant. Reverse strand.

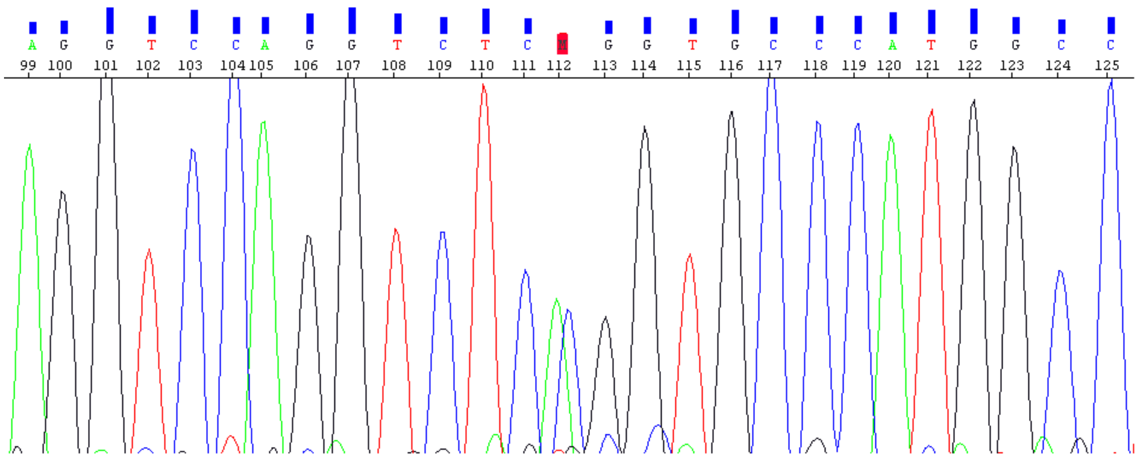

Supplementary figure S2: Integrative genome viewer of *MYBPC3* p.Gly263Ter variant. Reverse strand.

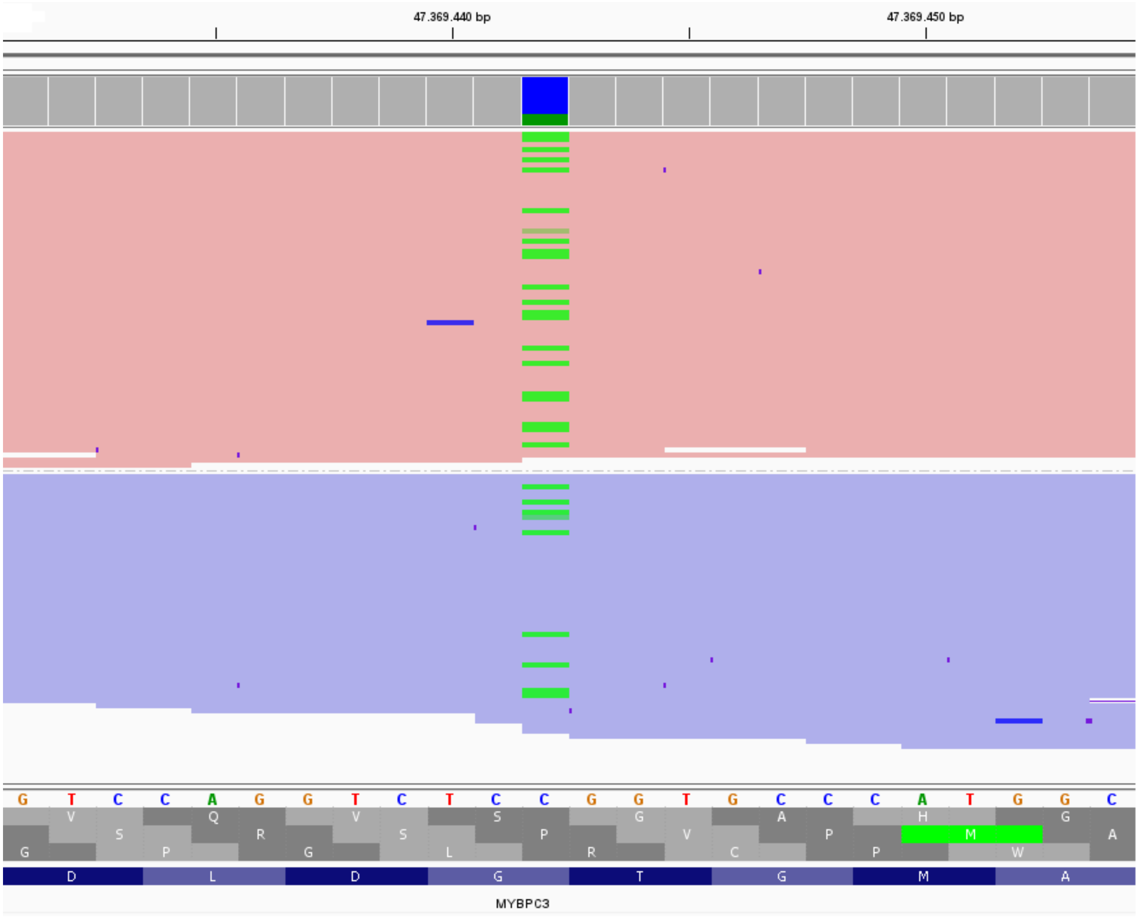

Supplement: Supplementary file 1 [file life-12-01346-s001.zip › life-1852695-supplementary.pdf]
